# What’s in a name? From “fluctuation fit” to “conformational selection”: rediscovery of a concept

**DOI:** 10.1007/s40656-021-00442-2

**Published:** 2021-07-09

**Authors:** Ferenc Orosz, Beáta G. Vértessy

**Affiliations:** 1 Institute of Enzymology, Research Centre for Natural Sciences, Magyar tudósok körútja 2, Budapest, 1117 Hungary; 2 Department of Applied Biotechnology and Food Sciences, Budapest University of Technology and Economics, Budapest, 1111 Hungary

**Keywords:** Fluctuation fit, Conformational selection, Induced fit, Lysenkoism, Brunó F. Straub, Gertrud Szabolcsi, Multiple discoveries

## Abstract

Rediscoveries are not rare in biology. A recent example is the re-birth of the "fluctuation fit" concept developed by F. B. Straub and G. Szabolcsi in the sixties of the last century, under various names, the most popular of which is the "conformational selection". This theory offers an alternative to the "induced fit" concept by Koshland for the interpretation of the mechanism of protein—ligand interactions. A central question is whether the ligand induces a conformational change (as described by the induced fit model) or rather selects and stabilizes a complementary conformation from a pre-existing equilibrium of various states of the protein (according to the fluctuation fit/conformational selection model). Straub and Szabolcsi’s role and the factors hindering the spread of the fluctuation fit theory are discussed in the context of the history of the Hungarian biology in the 1950s and 1960s.

**Supplementary Information:**

The online version contains supplementary material available at 10.1007/s40656-021-00442-2.

## Prologue^1^

Rediscoveries (or ‘rediscoveries’) are not rare in biology (Wheatley, 2018). Mendel’s Laws of Inheritance, the most famous relevant case, were forgotten for several decades after being published in 1865, then were ‘rediscovered’ independently by Carl Correns, Erich Tschermak and Hugo de Vries in 1900 (Harwood, 2000; Wolfe, 2012).^2^ Another famous case is the rediscovery of the sarcoplasmic reticulum that was first observed in 1902 by Emilio Veratti and rediscovered by electron microscopic studies in the 1960s (Mazzarello et al., 2003). Rediscoveries attest to the high significance of topics that necessarily re-emerge due to their crucial importance, even after long periods of “dormancy”. A similar situation seems to have occurred in the case of the concept termed “conformational selection”, considered as a “new molecular recognition paradigm” (Boehr et al., 2009).

## What is a rediscovery?

Rediscovery is a special case of a wider phenomenon referred to as “multiple independent discovery” by science historians, i.e., similar discoveries made by scientists working independently of each other (Garfield, 1980a). “*Sometimes the discoveries are simultaneous or almost so; sometimes a scientist will make a new discovery which, unknown to him, somebody else has made years before*.” (Merton, 1963, 237). He believed that multiple discoveries are rather the rule than the exception. It was explained by the social determination by the scientific *Zeitgeist.* According to this view, a discovery is inevitable when enough knowledge accumulates in “*man’s cultural store*” (Merton, 1963) and it is likely that more than one scientist achieves it independently (Stokes, 1986). This theory does not explain the special case of rediscoveries when there is a significant time interval (years or decades) between the multiple discoveries. As Simonton (1979, 1604) suggested, this kind of “premature” discoveries are due to the creativity of great scientists and these geniuses “*posses abilities, personalities and backgrounds that set them apart from their colleagues*”. The question arises why these premature discoveries are often (almost) forgotten.

However, we agree with the suggestion by Cole (1970) who considered premature discoveries as special subsets of the general phenomenon of *delayed recognition*, i.e., significant discoveries are unused or unappreciated until they are rediscovered years later. In some case, this process may be gradual and in others it is sudden (Garfield, 1980b). This general approach allows us to discuss rediscovery and “rediscovery’ in a unified way. From this point of view, it is irrelevant whether the original discovery was really rediscovered independently, was pulled out from the library drawer (‘rediscovery’) or was simply not used for a while.

So, what are the causes of this phenomenon? We consider 3 reasons: (i) conservatism in science; (ii) premature discovery from technical/methodological point of view; (iii) communication problems. The first and second points are interconnected while the third one raises an independent point of view.

Cole itself, who introduced the term delayed recognition, thought that the main reason is conservatism in science. In accordance with the then relatively fresh theory by Kuhn (1962), he said that scientists stick to their existing paradigms and resist new ideas that do not fit in (Cole, 1970). “*Skepticism about new ideas until they have been fully developed and adequately demonstrated fall well within the approved value system of scientists”* (Cole, 1970, 302). Paradigms are “*universally recognized scientific achievements that, for a time, provide model problems and solutions for a community of practitioners*” (Kuhn, 1962, 5). It should be noted that Cole found that this conservatism does not mean a kind of social one, i.e., not the low position of the discoverer/author in scientific hierarchy caused the delay, rather the inherent conservatism of science (Cole, 1970). It means that if a well-known recognised scientist comes up with an unusual idea or findings, he has no better chance to be recognised than a younger one.

The second possibility is that “*a paper will initially be ignored if its content cannot be extended* *experimentally to prevailing knowledge for technical reasons*” (Garfield, 1980b, 7). A new scientific model can be successful if it “*opens up or extends a domain of investigation by suggesting and generating new targets and thereby prompting and enabling new forms of empirical investigation*” (Peschard, 2011, 335). As experimental scientists, we consider this the most important factor from the point of view of practice, and it is also relevant for the case discussed in this paper. According to Knuuttila and Boon (2011) “*models give us useful knowledge through scientific practice, ( …) an ‘artefactual’ approach to modelling is required since traditional, representational approaches are either misleading or too minimal depending on how representation is defined*”. If the validity of a model cannot be checked experimentally it will not be applied (and the paper will not be cited) until the technical development renders it possible. This point is connected with the previous one since a paradigm is determined not only by the views of the scientists but also by the available techniques and methodologies of the research field.

The communication theory of multiple discoveries was developed by Brannigan and Wanner (1983). They highlighted the significance of the time interval between the first and last instances of discoveries. “*The announcements of discoveries do not always meet with unqualified success*” (Brannigan & Wanner, 1983, 138). The reasons are many. The ‘announcement’ can be lost in the ocean of scientific discoveries. It fails to become widely known since it was not published at all or was published not in the right place; “*it was unavailable to others outside the language/cultural group in which it was recorded*”; “*it was announced in such obscure language* (e.g., Hungarian or even Russian) *that potential readers were disentitled”*. The common trait which unites these cases “*a failure in the communication of results*”. This point also played a significant role in the subject of the paper.

## Biochemical background: molecular recognition

**Fig. 1:**
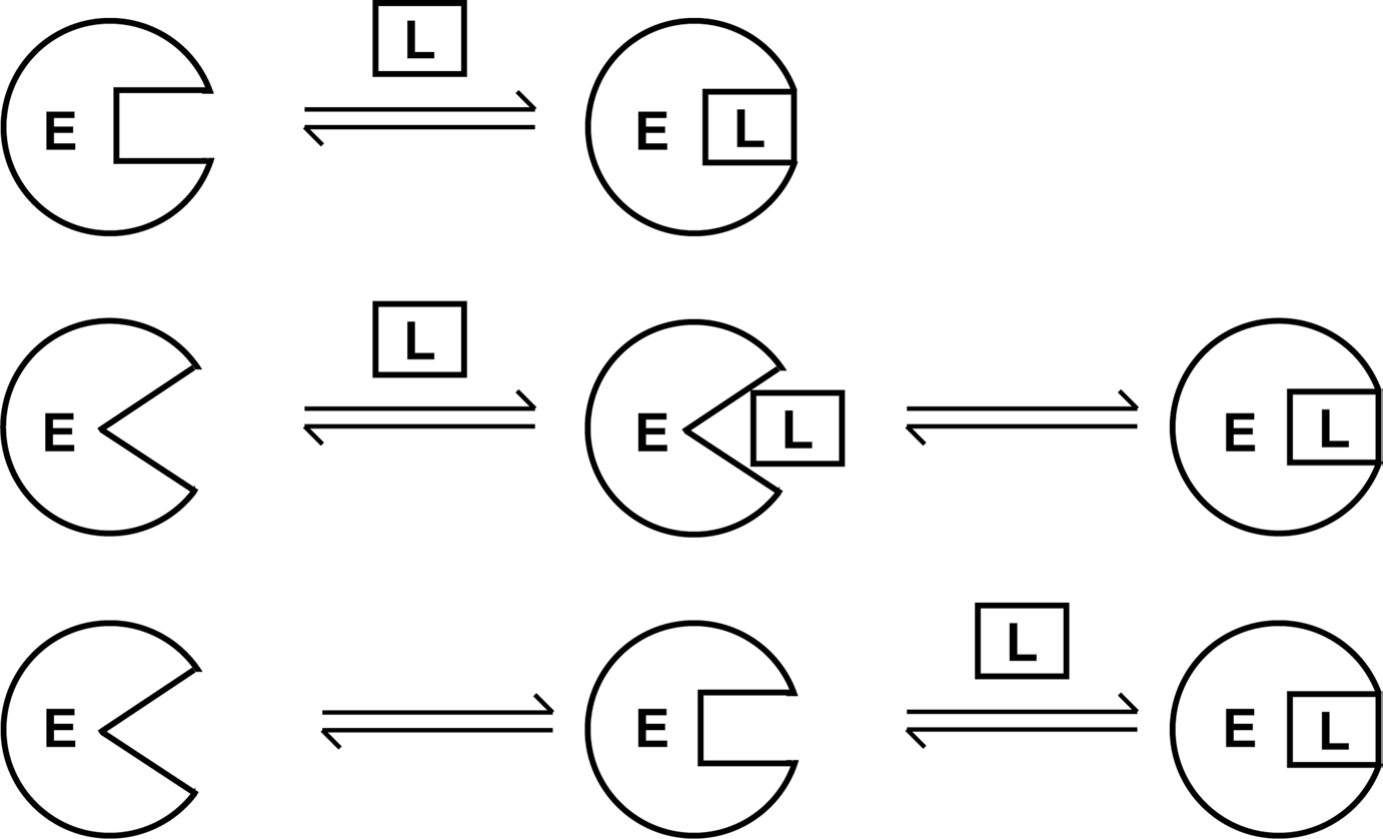
Models of protein–ligand (enzyme–substrate) binding: **a** “Lock-and-key” (Fischer, 1894): the enzyme is a rather rigid negative of the substrate, which has to fit into this negative to react. **b** Induced fit: the fit of the key (substrate) into the lock (enzyme) occurs only after the changes induced by the substrate itself; **c** Conformational selection (fluctuation fit) (E, enzyme; L, ligand): instead of a fit induced by the substrate, one particular form of the various states of the fluctuating enzyme molecule binds the substrate

**Fig. 2:**
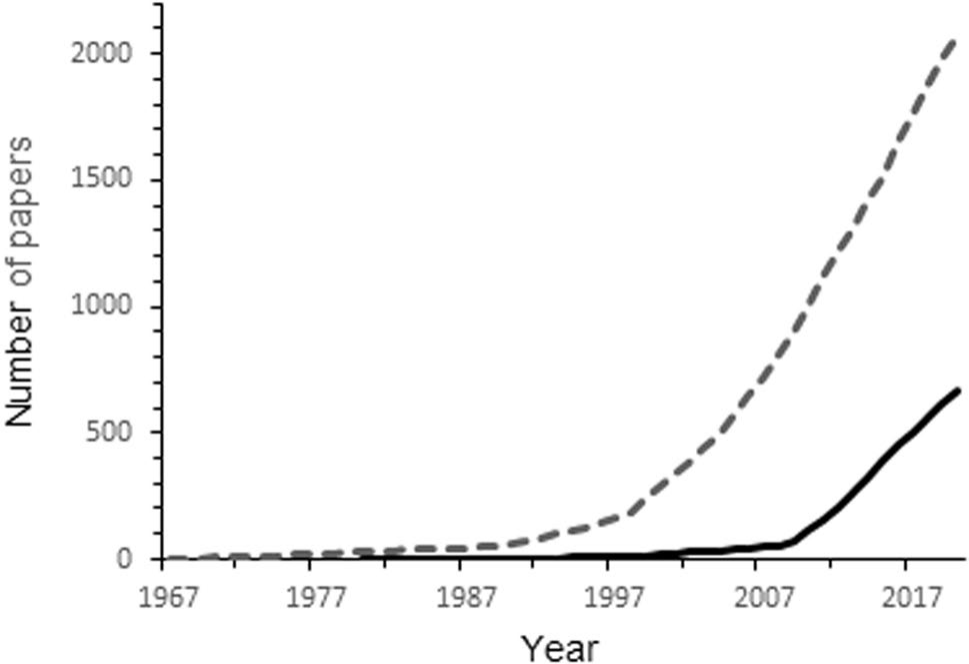
Cumulative number of papers using the term "conformational selection" (straight line) and induced fit” (dotted line), as listed in PubMed database (www.ncbi.nlm.nih.gov/pubmed; 26 August, 2020)

The binding of a ligand molecule to a protein (or other macromolecule) is often accompanied by conformational changes of the protein. A central question is whether the ligand induces the conformational change or rather selects and stabilizes a complementary conformation from a pre-existing equilibrium of various states of the protein (Weikl et al. 2009). The first one is the widely applied induced fit model (Koshland, 1958) (Fig. 1), which had great impact on our view of molecular recognition in the last half century (cf. Fig. 2); the second one is the conformational selection (Fig. 1). The essence of “conformational selection” is defined as a process during which the binding of two components (biological macromolecules to small molecular ligands or to each other) occurs in a milieu of *pre-existing equilibrium of multiple macromolecular conformations among which ligands can select the most fitting ones* (Boehr et al., 2009).

The appearance of the “conformational selection” term goes back to 40 years (Birdsall et al., 1980; Gronenborn et al., 1981); however, its widespread use started only two decades ago, when Nussinov and her co-workers published their first papers on the topic (Ma et al., 1999; Tsai et al., 1999a, 1999b). A decade ago, the papers published in this field started to increase exponentially, as shown in Fig. 2, based on a search of the PubMed database. Although the concept of induced fit developed by Koshland (1958) goes back to more than sixty years, the number of publications referring to it was jumping only in the very same time when the idea of conformational selection started to become popular (Fig. 2). The coincidence, in the cases of both models, was probably not by chance. Due to the development of the experimental techniques, first of all NMR methods, more and more dynamic structure of proteins complexed with ligands have been solved that needed interpretation.

**Fig. 3:**
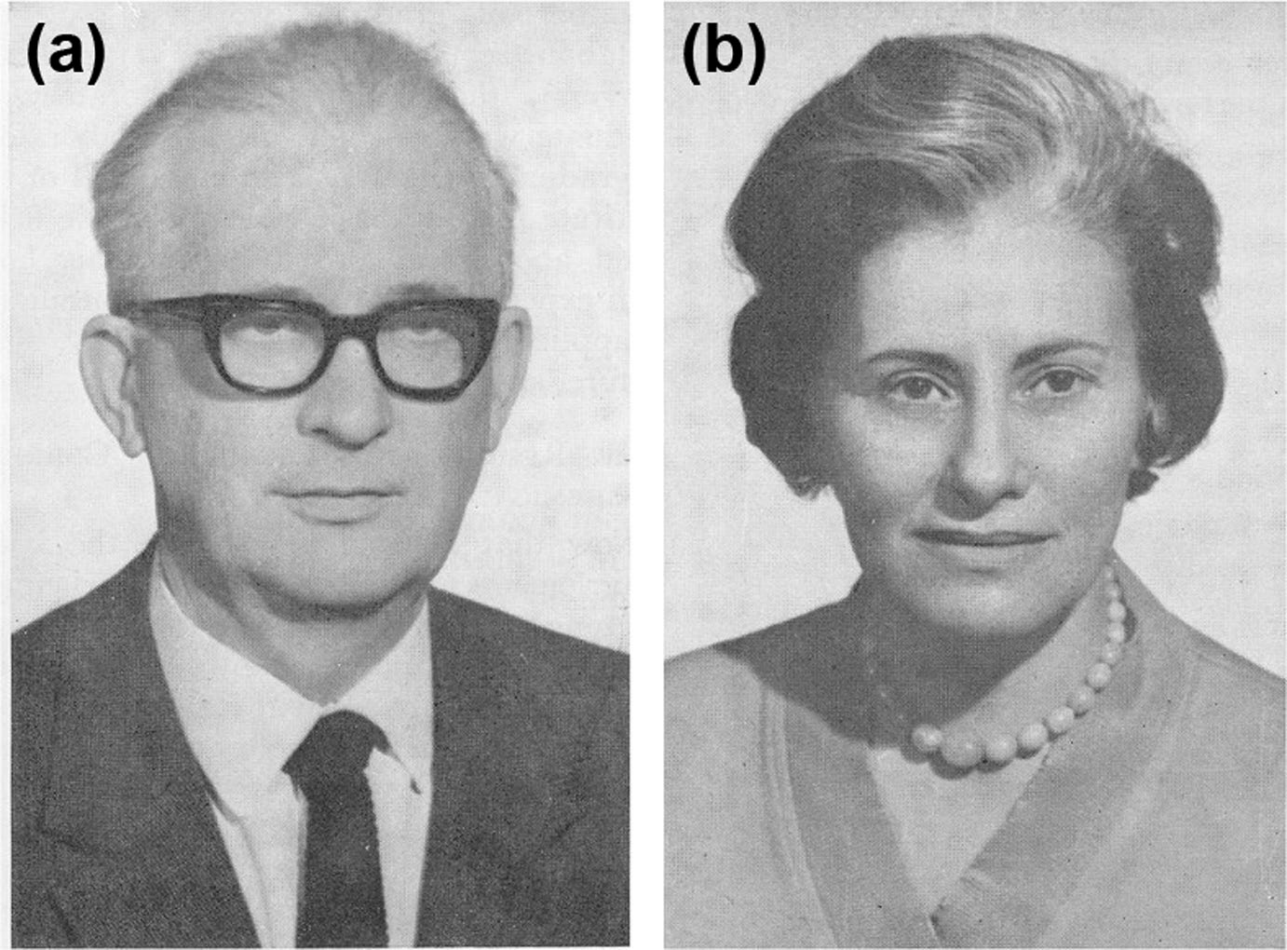
**a** Professor F. Brunó Straub (1914–1996) in 1972**. b** Professor Gertrud Szabolcsi (1922–1993) in 1972

Interestingly, an earlier, much similar, if not practically identical concept to conformational selection was already described in the sixties of the last century, although at that time it was termed as “*fluctuation fit*” (Straub & Szabolcsi, 1964). Obviously, the term “fit” indicates that the authors considered this mechanism as an alternative/complementary one for Koshland’s induced fit model.^3^ The term was coined by the Hungarian biochemist Brunó F. Straub (Fig. 3a), and his co-worker and soon to be wife, Gertrud Szabolcsi (Fig. 3b), director and deputy director of the Institute of Biochemistry of the Hungarian Academy of Sciences (HAS), respectively. The fundamental identity of the two concepts has been shown earlier (Vértessy & Orosz, 2011). A description of the conceptualization of the idea can also be found there, as well as in the work by Závodszky and Hajdú (2013). Straub (1967) himself also reported about it in a lecture. Thus, we do not go into biochemical details rather try to understand and explain why this early concept was almost forgotten.

When the popularity of conformational selection began at the millennium, neither Straub nor Szabolcsi were alive. As Wheatley (2018, 35) wrote: “*A discoverer of some phenomenon in science might have been dead for many decades, the work lost in the mists of time, or it might have been published in a low-impact journal and not visible*.” This generalized statement, with some modifications, fits well for the case discussed here, too.

## Some remarks on the authors of the 'fluctuation fit' paper in the frame of the history of Hungarian biochemistry

The roots of the Hungarian biochemical research reach back to the beginning of twentieth century. The early achievements attained their summit in 1937, when Albert Szent-Györgyi was awarded the Nobel Prize. Szent-Györgyi and his school at Szeged made many fundamental contributions to our understanding of cell respiration and the functioning of muscle (Friedrich, 1973). Brunó F. Straub (1914–1996) worked with Albert Szent-Györgyi from 1932 to 1945 at Szeged University, where he discovered actin (Straub, 1942).^4^ During this period, he spent two years (1937–39) in Cambridge as a post doc, in Prof. Keilin’s laboratory.

It was a promising start for Hungarian biochemistry and personally for Brunó F. Straub, but circumstances did not allow a fine development to follow. World War II ruined laboratories and scattered the members of Szent-Györgyi’s group. Szent-Györgyi himself went to the USA in 1947 and established a laboratory in Woods Hole (MA). Straub remained in Hungary and became a professor at Szeged University (1945), then in Budapest, at Pázmány Péter University (1948), and first a correspondent (1946), later a full member (1949) of the HAS. After the war, research had to be resumed mainly with young scientists and it took time for the newly created laboratories to come of age.

However, there was another reason which did not allow the straightforward development of Hungarian biological research after 1948. It was the so called the “year of the turn” in politics. However, the influence of Soviet occupation resulted in changes not only in politics but also in science. The pseudo-scientific concepts of “New Soviet (Michurin) Biology”, based on the Marxist-Leninist ideology rather than on scientific evidence (Borinskaya et al., 2019; Kolchinsky et al., 2017), were”exported” into the Eastern European countries under Soviet influence as well (DeJong-Lambert and Krementsov, 2012; 2017) and Hungary was not an exception (Igali, 2002; Müller, 2013; Palló & Müller, 2017). Besides the well-known “Lysenkoism”, which hindered mostly the work of agriculturists and geneticists, the New Cell Theory introduced by Olga Lepeshinskaya (1945) affected mostly cell biology and biochemistry. E.g., the Biology Division of HAS established a committee on Living Protein to investigate the New Cell Theory. It was led by the academician Imre Szörényi (Müller, 2013), director of the newly established Institute of Biochemistry of the HAS, which was the first Soviet type academy institute, founded in 1950 (Orosz, 2010, 2020). Straub himself was the vice-president of the committee; some young biochemists from Szörényi’s institute, including G. Szabolcsi, also participated in the work of the committee.^5^

Straub and other senior scientists (except if they were geneticist), although they had to declare their agreement with the “advanced” Soviet science, were more protected against this ideology based “science” than Szabolcsi and her young co-workers, who took their cues from Lepeshinskaya. They published two papers (Keleti et al., 1954, 1956) in a Hungarian journal in German, in which they reported on regeneration of yeast cells from completely homogenized yeast (*Saccharomyces cerevisiae*), in the same way as Lepeshinskaya (1945) claimed based on her experiments with hydras.^6^

Gertrud Szabolcsi (1922–1993), interestingly, was born in the same town (Nagyvárad) as Straub but they met each other only decades later. She was a Holocaust-survivor who was able to start her university studies only at age 23 when she returned from Auschwitz. In 1950, she was one of the founding members of the Institute of Biochemistry (today Institute of Enzymology) of the HAS where she served as a deputy director (1954–1972) until she married to Straub, director of the Institute from 1960 to 1986. She was the first woman biochemist elected to be a member of the HAS in 1967. Her research focused on the structure and function of enzymes. Although Straub and Szabolcsi worked in the same institute for 33 years and formed a couple for 21 years, they had never published a joint paper except the one discussed here.

As it is well known, 1956 is an important year in Hungarian (and European) history. Khrushchev’s speech at the 20th congress of the Soviet Communist Party in February and the Hungarian uprising in October had a significant impact on Hungarian *science*, too. On one hand, hundreds of thousands left Hungary for the “free world”, including many talented scientists, on the other hand, following of a period of terror, a slow “consolidation” started both in politics and a somewhat faster in scientific life. Publication restrictions disappeared. Between 1950 and 1956, it was forbidden to publish in "Western" newspapers. Lysenkoism and New Cell Theory ceased to be an “official” ideology in Hungary, although due to Khrushchev’s support, Lysenkoism was somewhat revitalized (Igali, 2002). By 1963, the political consolidation had become relatively wide-ranging. Following Khrushchev’s fall in 1964, Lysenko’s influence disappeared also in the Soviet Union (Kolchinsky et al., 2017).

However, for Szabolcsi and her colleagues this process was faster; maybe the reason was that they were not geneticists but biochemists and Lepeshinskaya’s ideas disappeared faster from Hungarian scientific life than other aspects of Michurin biology (Müller, 2013). The first author of both above mentioned papers, inspired by “New Cell Theory”, was Tamás Keleti, who published a scientific paper of real merit in Nature in 1957 (Antoni & Keleti, 1957), which was soon followed by Szabolcsi’s paper in the same prestigious journal (Elődi & Szabolcsi, 1959). Politically, both Szabolcsi and Keleti were faithful Communists, but it did not hinder them to do real science when it became ideologically free. (It should be added that for social and human scientists the ideological expectations ceased only decades later.) From the point of view of natural sciences, “Communism” lasted only about a good decade in Hungary. Straub itself, as a scientist and a science politician, had a significant role in the fact that, following the interlude of the fifties, Hungarian biology was able to return fast to the tradition hallmarked by Albert Szent-Györgyi. He became director of both the Institute of Biochemistry (later Enzymology) (1960–1986) and the Biological Research Centre (1970–1978) of the HAS. He served two terms as vice-president of the HAS (1967–1973; 1985–1988). In 1988–1989, he was the last president of the Presidential Council of the Hungarian People’s Republic, as the second biochemist following Ephraim Katchalsky-Katzir (Israel) becoming head of a state.

## The birth of the “fluctuation fit” concept

From 1960, Straub and Szabolcsi worked side by side, but separately, as the director and deputy director of the Institute of Enzymology of HAS. Straub devoted time to research in addition to his many scientific political activities and carried out protein renaturation experiments with enzymes containing SS-bridges (Venetianer & Straub, 1963, 1964) parallel to Anfinsen (Anfinsen 1961) who later received the Nobel Prize for his results in the field. Szabolcsi continued her work, started in 1956, on the relationship between the structure and function of enzymes (Szabolcsi & Biszku, 1961). Her name is associated with the introduction of proteolysis (protein degradation catalysed by proteases) in a study of the fine structure of enzymes and proteins (Biszku et al., 1964), and she has experimentally demonstrated that certain structural elements of enzymes have motility. In 1963, she wrote a dissertation for the title “Doctor of the Biological Sciences” (see later) in which, in addition to her own results, she discussed in detail the enzymological problems of the age, including Koshland's induced fit model. Although they did not work together, it is certain that Straub read the manuscript and even discussed it.^7^ In a relatively small institute with a familiar atmosphere, all the researchers were well acquainted with the work of their colleagues.

It was then that Straub was asked to contribute to the festive volume to be published in honour of Vladimir Aleksandrovich Engelhardt. The volume was published honouring Engelhardt on his 70th birthday in December 1964. Engelhardt (1894–1984), the father of the Soviet molecular biology, was the director of the Institute of Radiation and Physicochemical Biology of the Russian Academy of Sciences (from 1965 it was renamed as Institute of Molecular Biology).^8^ He discovered the aerobic re-synthesis of ATP, which is accompanied by cell respiration (oxidative phosphorylation). Together with his colleague, M. N. Liubimova, they discovered the enzymatic activity of myosin, and showed that ATP gives muscles the ability to function (Engelhardt & Liubimova, 1939).

The book was edited by Alexander Evseevich Braunstein, member of the Academy of Sciences of USSR, head of the biochemistry department of the institute led by Engelhardt.^9^ Braunstein (1902–1986), one of the “fathers of vitamin B6”, was the co-discoverer, along with Maria Kritzman, of enzymatic transamination (Braunstein & Kritzmann, 1937), and its dependence on vitamin B6 (Braunstein & Shemyakin, 1953).

Straub and Szabolcsi knew Braunstein personally. They met a few months earlier in Budapest. Braunstein was one of the reviewers of Szabolcsi’s thesis for the title “Doctor of the Biological Sciences” on 28th September 1964 (Anon. 1964b). (This title was the highest “rank” in the Soviet-type scientific system and a prerequisite for election to have a membership in the HAS.) It was a special event, it had never happened before that a foreign reviewer had been asked (Anon., 1964b). The choice was professionally justified. Szabolcsi’s dissertation title was “*Some directions in research on enzyme structure and function*” (Anon., 1970), while according to the Great Russian Encyclopedia “*a number of studies* (by Braunstein) *are devoted to studying the relationship between the structure and function of enzymes*”*.*^10^

Braunstein also visited the Institute of Biochemistry, of which Straub and Szabolcsi was then director and deputy director, respectively (Farkas, 1964). He stated that “*my chemistry institute has had a fruitful relationship with this research group* (Szabolcsi’s group) *for a long time”.* In fact, the series of joint publications of the Budapest Institute of Biochemistry and the Moscow Institute of Molecular Biology began the following year (Anon., 1970).^11^ Some of these publications served the. experimental verification of the fluctuation fit concept (Závodszky et al., 1966).

**Fig. 4:**
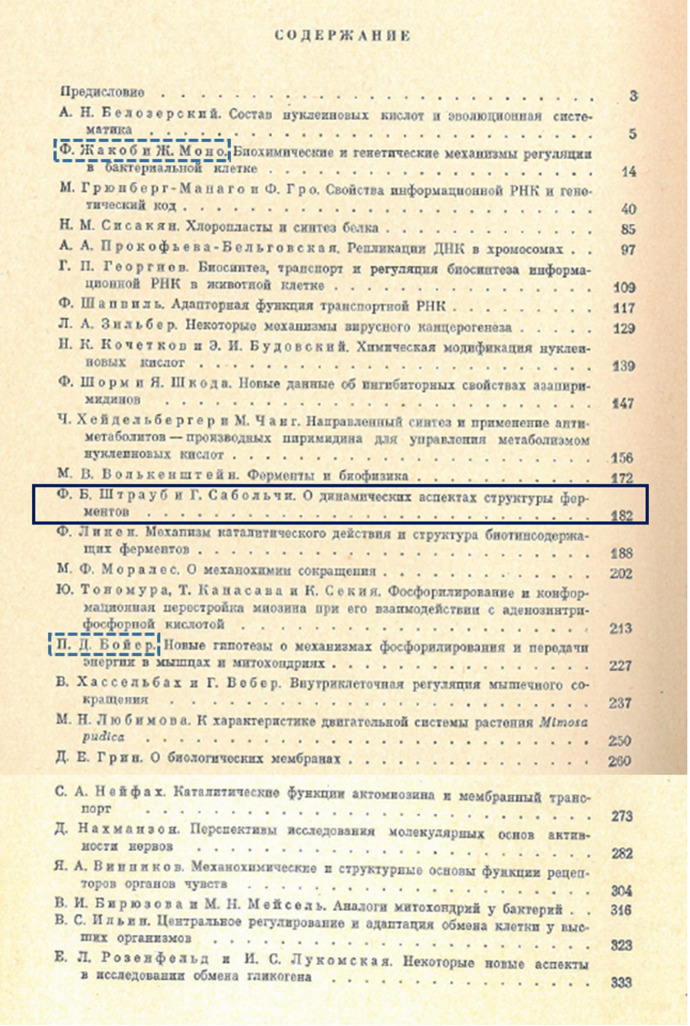
Table of content of Braunstein (1964). Box with full line indicate Straub’s and Szabolcsi’s paper (page 182); boxes with dotted lines indicate Jacob’s and Monod’s and Boyer’s publication, page 14 and 227, respectively

The collection edited by Braunstein contains 26 articles by Soviet and foreign authors, however, the papers of the foreign authors were translated into Russian. (For Table of content of the book see Fig. 4.) The vast majority of the articles are followed by an English summary, although in the case of French and German authors, the abstracts were written in their mother tongue. Today, it is difficult to understand why no English edition of the volume was also published. Most of the authors, including Straub, had achieved outstanding results on the topic of oxidative phosphorylation and muscle function (e. g., P. D. Boyer, M. F. Morales, D. Nachmansohn, Y. Tonomura), which was Engelhardt’s research interest. However, illustrious authors represented other fields of biochemistry/molecular biology as Belozhersky, another father of Soviet molecular biology and G. Weber, founder of Advances in Enzyme Regulation. They include François Jacob and Jacques Lucien Monod, who received the Nobel Prize in Medicine the following year, and Paul Delos Boyer, who was awarded the 1997 Nobel Prize in Chemistry. It is worth noting that Engelhardt’s birthday was in early December and Szabolcsi travelled to the USA for half a year in November (Anon., 1964a) where she worked in Boyer’s lab; cf. (Boyer et al, 1966.)

The main reason for Straub’s invitation was that he had previously worked in the same field (muscle function and the role of ATP in this) as Engelhardt. However, as his topic was different at the time, he decided not to write a “memoir” recalling his previous achievements, but to publish a theme that currently concerned him. A less rigorous, or no-peer review-going, book chapter offered an ideal opportunity to communicate a new theory.

Why did he write about fluctuation and why with Szabolcsi? At that time, the study of the relationships between the structure and function of enzymes was a defining topic not only for Szabolcsi, but—even as the legacy of the founding director, Szörényi—for the whole Institute of Enzymology of HAS. Deuterium—hydrogen exchange experiments of Linderstrom-Lang and Schellman (1959) had been known for several years, proving the flexibility of proteins. This recognition had a significant impact on the work being done at the institute. As a later director of the institute, Péter Závodszky, who was still starting his career at the time, wrote in his memorial paper (Závodszky & Hajdú, 2013, 265):

*The existence and functional importance of conformational flexibility was a basic concept in the interpretation of all experimental work on enzyme catalysis at the Institute of Enzymology, in Budapest since the early 60s, and this was atypical at those days for structural biochemists elsewhere.*

However, the flexibility of protein—including enzymes—was not linked to Koshland’s induced fit theory, which was published virtually at the same time, in 1958. Straub’s and Szabolcsi’s intuitive recognition on “fluctuation fit” preceded their age, and technical development later caught up with the idea.

Three years later, the concept received higher publicity when Straub presented it in a plenary lecture at the 7th IUB (International Union of Biochemistry) Congress, in Tokyo (the most important conference in the field of biochemistry arranged every 3 years) and was published in the Proceedings of the congress (Straub, 1967, 49).

*I have pointed out that a fluctuating model for an enzyme could be a good basis for the conformational changes occurring when enzyme binds its substrate or its product. Instead of a fit induced by the substrate, I would suggest a fluctuating enzyme molecule, one particular form of which is able to bind the substrate and other forms another ligand.*

However, importantly, neither of these publications used the term “fluctuation fit” but gave a detailed description of the mechanism. It was introduced only in 1969, five years after the publication of the original article. To add more international flavour, the terms induced fit and fluctuation fit were used in German (induzierte Anpassung and fluktuirende Anpassung, respectively), by Szabolcsi (1969). In this book-chapter not only the concept was explained in detail but, up to our knowledge, the term "fluctuation fit" was introduced although in German. The first English occurrence is in the same book (the preface of which was written by Straub) in a chapter by T. Keleti (1969), who used the term as a *terminus technicus* in the German text. This five-year-shift in the appearance of the concept and the proper name may be one of the reasons (beside the language problem) why the fluctuation fit concept did not become as popular as that of the induced fit. It is worth noting that Koshland (1958) introduced the theory and the terminology simultaneously in his original paper.

It is hard to understand why the Hungarian manuscript of the book was translated into German rather than English. Although German was preferred in the fifties by Hungarian biochemist but in the sixties, English was used more often than German, even in the journals printed in Hungary (Table 1). The last paper written in German by the members of the Institute of Biochemistry of HAS was published in 1966. Curiously, however, even in 1968, another German-language book was published by authors of the institute, at the same publisher, Akadémiai Kiadó (publisher of the HAS) (Dévényi & Gergely, 1968). Was it maybe the publisher’s policy that preferred the German language? English was an "imperialist" language, while German was also the language of the socialist German Democratic Republic. Moreover, the manuscript was practically completed in 1966, thus, as W.A. Schroeder (1970) pointed out, the translation would result in a significant delay. He also noted that “*A wider audience probably would have resulted had the translation been made into English*”. Today, it is no longer possible to determine what caused the delay. One of the authors of the volume, T. Keleti, already cited the book in an article submitted in March 1967 as a joint publication of Akadémiai Kiadó and Deuticke Verlag in Vienna (Keleti, 1967a). Finally, it was published only by the Hungarian publisher.

**Table 1:** Number of publications in the Institute of Biochemistry between 1951 and 1970

Year	Number of publications				
	Hungarian	German	English	Russian/Ukrainian	Total
1951	*2*				2
1952	2	*3*	1		6
1953	*1*				1
1954	3	**7**		2	12
1955	3	*4*	1	1	9
1956	8	*9*	5		22
1957	2	*6*	*2*		10
1958	3	3	*14*	1	21
1959	5	1	*6*		12
1960	2	2	*12*	1	17
1961	1	1	*10*		12
1962	1	1	*3*		5
1963	3		*6*		9
1964	6		*12*	1	19
1965	5	1	*10*	1	17
1966	2	3	*13*	3	21
1967	8		*19*	1	28
1968	3	1	*14*	1	19
1969	2	1	*9*		12
1970	4		*13*		17

Data are taken from Anon. (1970). Italic numbers indicate in which language most publications were published in a given year

It is worth noting in this connection that the notable book was based on the authors' doctoral dissertation—or, conversely, the chapters formed the basis of the dissertations (“Doctor of the Biological Sciences”). All four authors^12^ worked at the Institute of Biochemistry of the HAS. The preface was written by Straub. The first version of the manuscript of the book was ready in 1963. Szabolcsi, in her dissertation written in 1963 and defended in 1964, only discussed Koshland's induced fit theory, similarly to T. Keleti, who wrote his dissertation in 1964. The amended manuscript^13^ was completed in early 1967 (in Hungarian), in which both of them discussed the ‘Straub-Szabolcsi theory’,^14^ and the “fluctuation fit” name appeared the first time in Szabolcsi’s chapter—so far only in the manuscript.

## Further life of the concept

In another 4 years, the concept was popularised in English in an excellent review by Citri (1973) where the concept of “fluctuation fit” proposed by Straub and Szabolcsi was cited and summarized as an alternative theory to “induced fit”. Straightforward experimental proofs for co-existing molecular conformations in protein–ligand mixtures (Brocklehurst et al., 1983; Kilár & Závodszky, 1987; Polgár & Halász, 1978; Venyaminov et al., 1976; Závodszky et al., 1966) kept this concept alive until the advent of molecular dynamics simulations and high-resolution proton NMR measurements on proteins. These papers were published, with a few exceptions, by Hungarian and Russian researchers, but in English.

Table S1 summarizes the spectrum of diverse experimental techniques that allowed the early discovery and the further development of the “fluctuation fit” concept. We can observe that the specific term varies, however, the concept is the same and the different terms like “fluctuation fit” or e.g., “conformational selectivity” (Brocklehurst et al., 1983) are used in an interchangeable manner. Papers listed in Table S1 cite Straub’s and Szabolcsi’s work in the text (in some cases only indirectly or mistakenly) and the concept of the term “fluctuation fit”. The most recent one has been published recently (Benitez‐Amaro et al., 2020).

Likely, due to the fact that the original Straub and Szabolcsi paper was published in Russian, the later studies, after 1989, switched to citing another paper by Straub (1964) published in the same year in English, which does not contain the idea of fluctuation fit (cf. Table S1). Interestingly, this switch seems to coincide with the fall of communism in Eastern Europe (1989). Other type of shifts can be observed in several cases also caused by the language difficulties. Brocklehurst met the term "fluctuation fit" in a paper byPolgár and Halász (1978),^15^ who cited Straub and Szabolcsi's original work. First, Brocklehurst et al. (1983) referred to this Russian language publication indirectly (" 'fluctuation fit' by Straub and Szabolcsi (1964; cited by Polgár, 1978)"), however, later, when they mentioned fluctuation fit (Brocklehurst, 1994; Mellor et al., 1993; Pinitglang et al., 1997), they cited only Polgár's and Halász’s paper.

Even when the new term of conformational selection came into use as a “new paradigm” around the turn of the century, some authors (Bursavich & Rich, 2002, 542) remembered the original concept, but cited Straub’s another work (Straub, 1964) mentioned above:

*Recently, stabilization of receptor conformational ensembles (*Ma et al., 1999; Tsai et al., 1999a*) has emerged to rationalize a range of ligand binding events without necessitating either the lock-and-key (cf. Fig. **1**) or induced fit mechanisms. This model assumes that macromolecules exist as multiple, equilibrating solution conformations that can be described by* *mechanical laws with standard statistical distributions. The process of ligand binding effectively shifts this equilibrium to the bound-receptor conformation from the statistical distribution of native conformations. In this view, ligands bind to the ensemble of pre-existing receptor conformations. Productive binding shifts the overall dynamic equilibrium to stabilize the bound receptor conformation. This concept of conformationally mobile receptors (and ligands) is not new, but arose shortly after the discovery of modern conformational analysis. Almost 40 years ago, Straud (sic !) stated (*Straub, 1964*) 'the conformation of an enzyme in solution is regarded to be a statistical average of a number of conformations, the protein structure oscillating between these conformations’*.

Thus, the fluctuation fit concept, but not the term, was still present in the literature as an “underground stream” and formed an alternative basis for experimental design and interpretation using sophisticated methodologies within the field of enzyme structure and mechanism.

As can be seen from the table, the original paper by Straub and Szabolcsi (1964) was not cited between 1989 and 2010! Then an extensive “campaign” by Hungarian researchers (Csermely et al., 2010; Vértessy & Orosz, 2011; Závodszky & Hajdú, 2013) went back to the source and placed back the basic work on the map. Subsequently, others have articulated the identity of fluctuation fit and conformational selection: “*We consider fluctuation fit and conformational selection to be equivalent*” (Fenwick et al., 2011, 1344), and this has been recognized also by the leading fighters of conformational selection. It seems obvious that they simply did not know either the term or the original concept. The author who considered conformational selection as a ‘‘*new molecular recognition paradigm*’’ (Boehr et al., 2009), three years later wrote (Boehr, 2012, 175):

*Although induced fit has been the concept favored by biochemistry textbooks for decades, the conformational selection model, in the guise of ‘fluctuation fit’, has almost as much history (Straub & Szabolcsi* 1964; Vértessy & Orosz, 2011*).*

Finally, we cannot resist inserting a longer paragraph here from Mannige’s article (Mannige, 2014, 135) that even we could have written:

*Conformational selection” is the same as “fluctuation fit” from the 1960’s. It is noteworthy that this dynamic mode of protein binding is not as new as it might seem (…) shortly after “induced fit” contended for the textbooks (*Koshland, 1958*), Straub and colleague synthesized a prototype of today’s conformational selection, which he then called the fluctuation fit model (*Straub, 1967; Straub & Szabolcsi, 1964*). The mechanism of fluctuation fit, unfortunately, could not be experimentally resolved from that of induced fit at the time of its introduction (Orosz and Vértessy 1991), and so these concepts were left dormant until further and more discerning experimental and computational techniques emerged (Orosz and Vértessy 1991). From Straub till today, this dynamic mode of binding has gathered many monikers some of which are: “fluctuation fit”, “conformational selection”, “conformational selectivity”, “population shift”, “selected fit”, “stabilization of conformational ensembles”, and “preexisting equilibrium” (reviewed in (Orosz and Vértessy 1991)).*

The sentence “*The mechanism of fluctuation fit, unfortunately, could not be experimentally resolved from that of induced fit at the time of its introduction”* has extreme importance considering the recent story. “*The worth of a model depends on the difference it makes in epistemic space with respect to the investigation of scientifically significant problems*” (Ankeny et al., 2011, 306). The opportunity to differentiate only came decades later, with the advent of experimental techniques such as NMR relaxation (Boehr et al., 2006) and single-molecule fluorescence (Michalet et al., 2006) studies. Beside the “visibility problems” of the original publication (“*failure in the communication of results*”), it was the reason of the long dormancy of the fluctuation fit model.

We, the authors, became acquainted with the “fluctuation fit” concept as we started our Ph.D. studies at the Institute of Enzymology where Straub was the director. Our experiments in enzymology concerning structure and function relationships were evaluated in the light of the induced fit and fluctuation fit concepts. Both concepts seemed to us as textbook axioms and therefore we discussed these alternative mechanisms without citing any references in our published studies (cf. e.g., Vértessy et al., 1991).

## Epilogue

By the rise of systems biology and data-driven research, significant portions of the treasure cave of the first “golden age” of enzymology and mechanistic investigations became, somewhat unfortunately, akin to a “Sleeping Beauty” (van Raan, 2004) warehouse of information. There is therefore a substantial need for adequate princes to mine and dredge this database so that the research community may live happily and knowingly ever after.

Different phraseology describing the same ideas or findings is quite frequent not just in biology but in other fields of science as well. In some cases, when scientists realize the equivalence of multiple terms, some unifying efforts may be exercised, but these are usually not uniformly accepted. A current example is provided by the research into proteins with high levels of conformational freedom – these may be called either “intrinsically unstructured” (Wright & Dyson, 1999) or “intrinsically disordered” (Dunker et al., 2001) proteins. Although in the case discussed in the present paper, the question of terminology is concerted, it is straightforward to realize that the terms “fluctuation fit” and “conformational selection” describe the very same concept. It can be added that the latter term describes the phenomenon perhaps more precisely than the former one, however, the name “fluctuation fit” is as genial as the concept itself. Anyway, as noted by the Great Bard:

*What's in a name? that which we call a rose, By any other name would smell as sweet*.” (William Shakespeare in Romeo and Juliet (II, ii, 1-2)).

## Supplementary Information

Below is the link to the electronic supplementary material.

Supplementary file1 (DOCX 19 kb)

## Footnotes

1 Abbreviation: HAS – Hungarian Academy of Sciences.

2 Today, it is generally accepted that publishing their papers in 1900 they had known Mendel’s work (e.g., Simunek et al., 2011). However, some of their experimental results corroborating Mendel’s findings had been achieved years earlier independently (e.g., Stamhuis et al., 1999).

3 45 years later Weikl and von Deuster (2009) suggested the name of “selected-fit”, but it could not displace the use of “conformational selection”.

4 The aftermath of this discovery is also an interesting story which demonstrates how history influenced science in Hungary. For details see the papers written by Mommaerts (1992), Szent-Györgyi (1996) and Perry (2003).).

5 Protocols of the Academy Committee on Living Protein (Archives of HAS, Biológiai Osztály Iratai, 108/14/1. The authors thanks to Dr. Diána Háy, director of the Archives of HAS, for providing the document.

6 Lepeshinskaya (1945) Chapter XXII. pp. 157–170. The preface of the book was written by Lysenko. In German translation: Lepeschinskaja, O. B. (1952) 22. Kapitel. Die Entwicklng der aus Zellen der Hydra gewonnenen lebenden Materie und ihre Fortbildung, pp 191–207.

7 The authors are well acquainted with the institutional practice, which was alive even in the 1980s, that Straub as a director—and often Szabolcsi—read the dissertations from all members of the institute and formed an opinion about them.

8 Four years after Engelhardt's death, in 1988, the institute took on Engelhardt's name.

9 The executive editor of the publication was A. E. Braunstein; further editors were A. N. Nesmeyanov (former President of the Soviet Academy of Sciences, 1951–61), and I. E. Tamm (Nobel-laureate in physics, 1958) academicians as well as A. A. Baev and T. V. Venkstern.

10 https://bigenc.ru/biology/text/1882609

11 The first joint paper was by Polianovskiĭ and Telegdi (1965) on aspartate-glutamate transaminase. According to PubMed, Braunstein published eight papers with Polianovskiĭ, all of them on aminotransferases.

12 Tibor Dévényi, Pál Elődi, Tamás Keleti, Gertrud Szabolcsi.

13 The dissertations and the manuscripts can be found in the library of Institute of Enzymology, Research Centre for Natural Sciences, Budapest.

14 Keleti used also this term in his English language papers published in one of the journals of the HAS (Keleti 1967b, 1968). It suggests that although the Hungarian text of the book was ready in early 1967, some changes were probably introduced during the translation.

15 These authors worked in the institute directed by Straub.
