# Supplementary material for: What’s in a name? From “fluctuation fit” to “conformational selection”: rediscovery of a concept
Source: Hist Philos Life Sci. 2021 Jul 9;43(3):88. doi: 10.1007/s40656-021-00442-2 (PMC8270835; doi:10.1007/s40656-021-00442-2)
Supplement: Supplementary file 1 — Supplementary file1 (DOCX 19 kb) [file 40656_2021_442_MOESM1_ESM.docx]

**Table S1 Studies and reviews presenting the concept of fluctuation fit also under alternative terms**

| Publication | Method | Object of study | Specific term used instead of/ in addition to fluctuation fit | Reference cited |
| --- | --- | --- | --- | --- |
| Straub and Szabolcsi 1964 | SH reactivity | GAPDH |  | - |
| Závodszky et al. 1966 | H-D exchange | GAPDH |  | Straub and Szabolcsi 1964 |
| Straub 1967 | SH reactivity | GAPDH, RNase |  | Straub and Szabolcsi 1964 |
| Keleti 1967, 1968, 1969 | theoretical work | - | Straub-Szabolcsi theory | Straub and Szabolcsi 1964 |
| Szabolcsi 1969 | review | review | fluktuirenden Anpassung | Straub and Szabolcsi 1964 |
| Libor and Elődi 1970 | enzyme kinetics | GAPDH |  | Straub and Szabolcsi 1964 |
| Citri 1973 | review | review | conformative response, conformational adaptibility | Straub 1964, Szabolcsi 1969 |
| Telegdi and Straub 1973 | enzyme kinetics | amylase |  | Straub and Szabolcsi 1964 |
| Simon et al. 1974 | small angle X-ray scattering | amylase |  | Straub and Szabolcsi 1964 |
| Vas and Boross 1974 | SH reactivity (kinetic analysis) | GAPDH |  | Straub and Szabolcsi 1964, Szabolcsi 1969 |
| Buc-Carbon and Buc 1975 | relaxation studies | phosphorylase b | selective role of the ligand | Straub and Szabolcsi 1964 |
| Keleti et al. 1976 | review |  |  | Straub and Szabolcsi 1964 |
| Venyaminov et al. 1976 | H-D exchange | immunoglobulin G1 |  | Straub and Szabolcsi 1964 |
| Venyaminov et al. 1976 | SH reactivity | papain |  | Straub and Szabolcsi 1964 |
| Keleti et al. 1977 | review |  |  | Straub and Szabolcsi 1964 |
| Polgár and Halász 1978 | enzyme kinetic | papain |  | Straub and Szabolcsi 1964 |
| Ptytsin 1978 | review |  |  | Straub and Szabolcsi 1964 |
| Welch and Keleti 1981 | theoretical work |  |  | Straub and Szabolcsi 1964 |
| Brocklehurst et al. 1983 | enzyme kinetics | papain | conformational selectivity | Straub and Szabolcsi 1964, Polgár and Halász 1978 |
| Damjanovich et al. 1983 | theoretical work |  |  | Straub and Szabolcsi 1964 |
| Kilár et al. 1985 | small angle X-ray scattering | immunoglobulin G1 |  | Straub and Szabolcsi 1964 |
| Kilár and Závodszky 1987 | H-D exchange | immunoglobulin G1 |  | Straub and Szabolcsi 1964 |
| **Szabó et al. 1989** | theoretical work |  |  | **Straub and Szabolcsi 1964** |
| Vértessy et al. 1991 | enzyme kinetics, fluorescence anisotropy | aldolase |  | - |
| Mellor et al. 1993 | stopped-flow kinetics | papain | conformational selectivity | Polgár and Halász 1978 |
| Brocklehurst 1994 | review | review | conformational selectivity | Polgár and Halász 1978 |
| Pinitglang et al. 1997 | stopped-flow kinetics | cystein proteinases | conformational selectivity | Polgár and Halász 1978 |
| Závodszky et al. 1998 | CD, FT-IR | 3-isopropylmalate dehydrogenase |  | Straub 1964 |
| Bursavich and Rich 2002 | review | review | stabilization of conformational ensembles | Straub 1964 |
| Matsuura et al. 2002 | X-ray | lysozyme |  | Straub 1964 |
| Csermely 2008 | review | review | conformational selection | Straub 1964 |
| **Csermely et al. 2010** | review | review | conformational selection | **Straub and Szabolcsi 1964** |
| Vértessy and Orosz 2011 | review | review | conformational selection |  |
| Fenwick et al. 2011 | review | review | conformational selection | Straub and Szabolcsi 1964 |
| Boehr 2012 | NMR | splicing factor U2AF | conformational selection | Straub and Szabolcsi 1964 |
| Gáspár and Csermely 2012 | theoretical work | biological networks |  | Straub and Szabolcsi 1964 |
| Csermely et al. 2013 | review | drug discovery |  | Straub and Szabolcsi 1964 |
| Hegedűs et al. 2013 | network methods | ABC transformers |  | Straub and Szabolcsi 1964 |
| Kastritis and Bonvin 2013 | review | proteins |  | Straub and Szabolcsi 1964 |
| Szilágyi et al. 2013 | network methods | drug research |  | Straub and Szabolcsi 1964 |
| Pons et al. 2013 | docking | ubiquitin | conformational selection | Straub and Szabolcsi 1964 |
| Závodszky and Hajdú 2013 | review | review | conformational selection | Straub and Szabolcsi 1964 |
| Mannige 2014 | review | review | conformational selection | Straub and Szabolcsi 1964  Straub 1967 |
| Buonfiglio et al. 2015 | review | drug design |  | Straub and Szabolcsi 1964 |
| Shukla et al. 2015 | theoretical work | review | conformational selection | Straub and Szabolcsi 1964 |
| Szöllősi et al. 2016 | in silico docking |  | conformational selection | Straub and Szabolcsi 1964 |
| Pallara et al. 2016 | theoretical work | protein-protein interaction | conformational selection | Straub and Szabolcsi 1964 |
| Gupta 2018 | review | drug design | conformational selection | Straub and Szabolcsi 1964 |
| Benitez-Amaro et al. 2020 | molecular modelling | low density lipoprotein |  | Straub and Szabolcsi 1964 |

Bold letters indicate that the paper written by Straub and Szabolcsi (1964) was not cited at all between 1989 and 2010.

**References (additional)**

Buc-Carbon M.H. & Buc H.C., (1975). Quaternary changes of rabbit-muscle glycogen phosphorylase b at low temperature: relaxation studies and titration of sulfhydryl groups. *European Journal of Biochemistry* 52, 575-588.

Buonfiglio R., Recanatini M., & Masetti M. (2015) Protein flexibility in drug discovery: from theory to computation. *ChemMedChem* 10, 1141-1148.

Csermely P. (2008). Creative elements: network-based predictions of active centres in proteins and cellular and social networks. *Trends in Biochemical Sciences* 33, 569-576.

Csermely, P., Korcsmáros, T., Kiss, H.J., London, G. & Nussinov, R. (2013) Structure and dynamics of molecular networks: A novel paradigm of drug discovery: A comprehensive review. *Pharmacology and Therapeutics* 138, 333-408.

Damjanovich, S., Somogyi, B. & Welch, G.R. (1983) Protein fluctuation and enzyme activity. *Journal of Theoretical Biology* 105, 25-33.

Gáspár, M.E. & Csermely, P. (2012) Rigidity and flexibility of biological networks. *Briefings in Functional Genomics* 11, 443-456.

Gupta S.P. (2018) Protein flexibility: A challenging issue of drug discovery. *Current Chemical Biology* 12, 3-13.

Hegedűs, T., Gyimesi, G., Gáspár, M.E., Szalay, K.Z., Gangal, R. & Csermely, P. (2013) Potential application of network descriptions for understanding conformational changes and protonation states of ABC transporters *Current Pharmaceutical Design* 19, 4155-4172.

Kastritis, P.L. & Bonvin, A.M.J.J. (2013) On the binding affinity of macromolecular interactions: Daring to ask why proteins interact. *Journal of the Royal Society Interface* 10, Article number 20120835

Keleti, T., Batke, J., Ovádi, J., Jancsik, V. & Bartha, F. (1977) Macromolecular interactions in enzyme regulation. *Advances in Enzyme Regulation* 15(C), 233-265.

Keleti, T., Ovádi, J. & Batke, J. (1976) Catalysts and enzymes. (The thermodynamic and kinetic basis of enzyme regulation.) *Journal of Molecular Catalysis* 1, 173-200.

Kilár, F., Simon, I., Lakatos, S., Vonderviszt, F., Medgyesi, G.A., Závodszky, P. (1985) Conformation of human IgG subclasses in solution: Small‐angle X‐ray scattering and hydrodynamic studies. *European Journal of Biochemistry* 147, 17-25.

Libor, S. & Elödi, P. (1970) Selective reaction of tyrosyl side chains with iodine in d‐glyceraldehyde 3‐phosphate dehydrogenase: 1. study of the differential formation of monoiodo and diiodo derivatives. *European Journal of Biochemistry* 12, 336-344.

Matsuura A., Yao M., Aizawa T., Koganesawa N., Masaki K., Miyazawa M., Demura M., Tanaka I., Kawano K. & Nitta K. (2002). Structural analysis of an insect lysozyme exhibiting catalytic efficiency at low temperatures. *Biochemistry* 41, 12086-12092.

Pallara C., Rueda M., Abagyan R. & Fernández-Recio J. (2016) Conformational heterogeneity of unbound proteins enhances recognition in protein-protein encounters. *Journal of Chemical* *Theory and Computation* 12, 3236-3249.

Pons, C., Fenwick, R.B., Esteban-Martín, S., Salvatella, X. & Fernandez-Recio, J. (2013) Validated conformational ensembles are key for the successful prediction of protein complexes. *Journal of Chemical Theory and Computation* 9, 1830-1837.

Ptitsyn, O.B. (1978) Inter-domain mobility in proteins and its probable functional role. *FEBS Letters* 93, 1-4.

Shukla D., Hernández C.X., Weber J.K. & Pande V.S. (2015) Markov state models provide insights into dynamic modulation of protein function. *Accounts of Chemical Research* 48, 414-422.

Simon I., Móra S. & Elődi P. (1974) Studies on the active center of pancreatic amylase. II. Small angle x-ray scattering investigations. *Molecular and Cellular Biochemistry* 4, 211-216.

Telegdi, M. & Straub, F.B. (1973) Study of correlation between structural motility and reactivity of SH groups in α-amylase. *BBA – Enzymology* 321, 210-219.

Szabó, G., Somogyi, B. & Damjanovich, S. (1989) Macromolecular fluctuation and biological function. *Journal of Molecular Liquids* 42C, 59-70.

Szilágyi, A., Nussinov, R. & Csermely, P. (2013) Allo-network drugs: Extension of the allosteric drug concept to protein- protein interaction and signaling networks. *Current Topics in Medicinal Chemistry* 13, 64-77.

Szöllősi D., Erdei Á., Gyimesi G., Magyar C. & Hegedűs T. (2016) Access path to the ligand binding pocket may play a role in xenobiotics selection by AhR. *PLoS One* 11, e0146066.

Vas M. & Boross L. (1974). An approach for the determination of equilibrium constant of structural motility. Characterization of the fluctuational motion around residue Cys-153 of D-glyceraldehyde-3-phosphate dehydrogenase. *European Journal of Biochemistry* 43, 237-244.

Welch, G.R. & Keleti, T. (1981) On the "cytosociology" of enzyme action in vivo: A novel thermodynamic correlate of biological evolution. *Journal of Theoretical Biology* 93, 701-735

Závodszky P., Kardos J., Svingor A. & Petsko G.A. (1998). Adjustment of conformational flexibility is a key event in the thermal adaptation of proteins. *Proceedings of the National Academy of Sciences of the United States of America* 95, 7406-7411.
